# Learning Curve of Robotic Right Hemicolectomy

**DOI:** 10.1007/s11605-022-05343-8

**Published:** 2022-05-09

**Authors:** Bo Tang, Yahang Liang, Jun Shi, Taiyuan Li

**Affiliations:** Department of General Surgery, The First Affiliated Hospital of Nanchang University, Nanchang, 330006 Jiangxi China

**Keywords:** Robotic, Right hemicolectomy, Learning-curve, CUSUM, RA-CUSUM

## Introduction

After  the safety and feasibility of robotic right hemicolectomy is demonstrated ^1,2^, it is important to analyze the learning curve to determine how this technique can be taught to novices. However, studies focused on the learning curve of robotic right hemicolectomy are limited.

## Methods

The clinical records of consecutive patients who underwent robotic right colon cancer resection performed by a single surgeon between April 2015 and December 2018 in the First Affiliated Hospital of Nanchang University were retrospectively reviewed.

Cumulative sum (CUSUM) and risk-adjusted cumulative sum (RA-CUSUM) ^3,4^ were applied to assess the learning curve of operation time and surgical failure (conversion, Clavien–Dindo (CD) ≥ grade III, harvested lymph nodes less than 12, R1 resection). Qualitative data were analyzed using the chi-square test or Fisher’s exact test, and quantitative data were analyzed using Student’s *t* test or the Mann–Whitney *U* test. *P* values of < 0.05 were considered statistically significant.

## Results

**Fig. 1:**
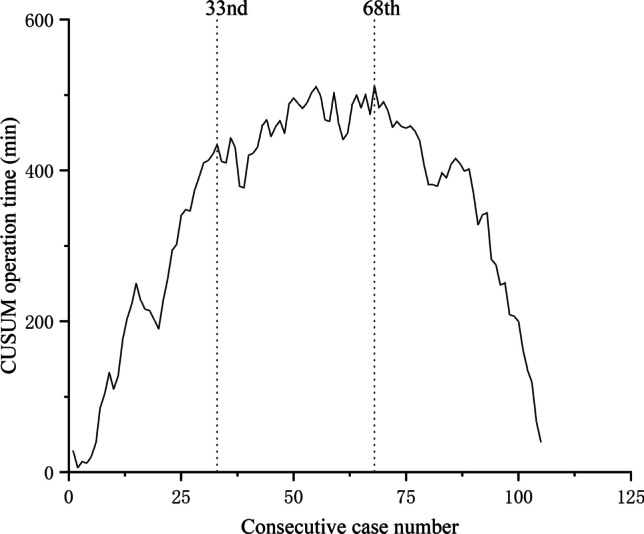
CUSUM for operation time

**Fig. 2:**
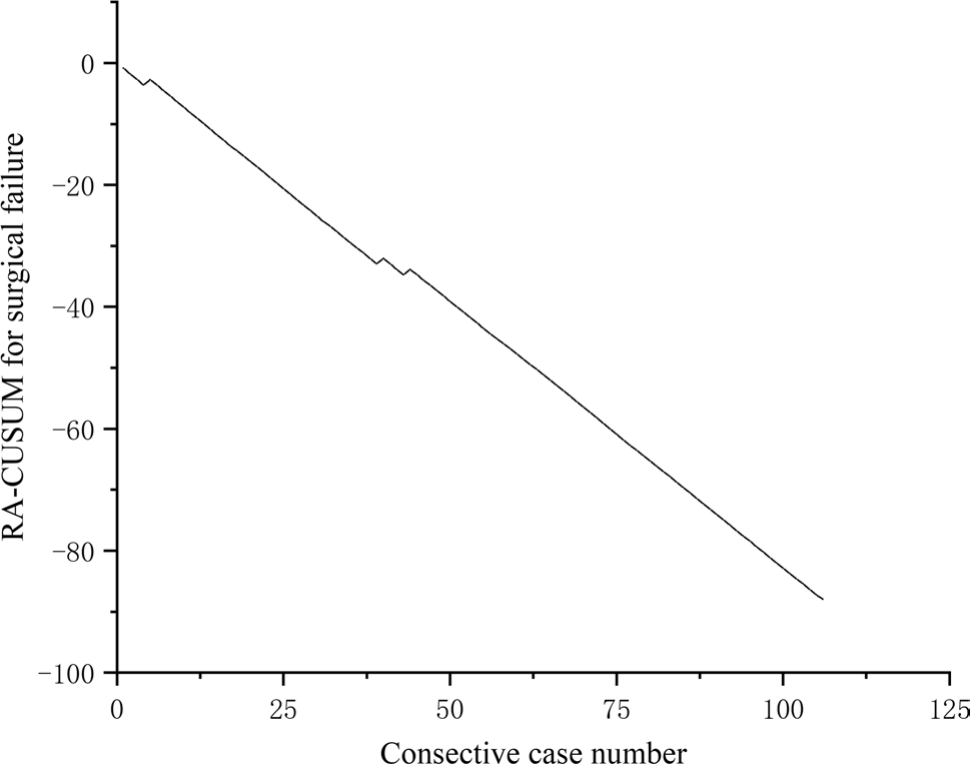
RA-CUSUM for surgical failure

A total of 106 patients were included. The learning curves of operation time and surgical failure are shown in Figs. 1 and 2.

The characteristics and perioperative outcomes of patients during different phases are presented in Table 1.

**Table 1:** Patient characteristics and perioperative outcomes between different phases

variable	Phase 1 (*n* = 33)	Phase 2 (*n* = 35)	Phase 3 (*n* = 38)	*P* value
Age (years)	61.9 ± 13.8	62.7 ± 12.6	60.6 ± 14.7	0.818
Sex				0.581
Man (%)	17 (51.5)	16 (45.7)	22 (57.9)	
Women (%)	16 (48.5)	19 (54.3)	16 (42.1)	
BMI (kg/m^2^)	21.7 ± 1.6	22.3 ± 2.5	21.9 ± 2.3	0.568
ASA (I/II/III, %)	14 (42.4)/17 (51.5)/2 (6.06)	20 (57.1)/13 (37.1)/2 (5.7)	25 (65.8)/11 (28.9)/2 (5.3)	0.389
CEA (ug/L)	3.2 (0.4–520.0)	8.39 (0.2–105.0)	6.54 (0.8–82.5)	0.582
CA19.9 (U/mL)	9.75 (3.1–500.6)	14.88 (0.6–184.0)	6.11 (0.6–171.8)	0.407
Operation time (min)	185.1 ± 19.6	177.1 ± 38.6	160.7 ± 36.2	0.007
Blood loss (mL)	171.7 ± 70.1	131.1 ± 17.4	127.9 ± 21.3	0.000
Postoperative hospital stays (d)	9.1 ± 4.9	9.0 ± 3.0	7.8 ± 1.6	0.207
Postoperative complications (%)	5 (15.2)	4 (11.4)	4 (10.5)	0.825
Anastomosis leakage (%)	1 (3.0)	1 (2.9)	0 (0)	
Intestinal obstruction (%)	1 (3.0)	1 (2.9)	1 (2.6)	
Wound infection (%)	1 (3.0)	1 (2.9)	1 (2.6)	
Pulmonary infection (%)	0 (0)	1 (2.9)	1 (2.6)	
Intra-abdominal infections (%)	1 (3.0)	0 (0)	1 (2.6)	
Bleeding (%)				
Reoperation (%)	1 (3.0)	0 (0)	0 (0)	0.565
Tumor size (mm)	31.0 ± 11.9	37.3 ± 10.1	33.8 ± 11.4	0.068
Differentiation (high/moderate/low, %)	0 (0)/32 (97.0)/1 (0.3)	1 (2.9)/32 (91.4)/2 (5.7)	2 (5.3)/31 (81.6)/5 (13.2)	0.303
Resected lymph nodes	18.9 ± 4.7	19.9 ± 7.5	20.6 ± 5.9	0.520
TNM stage				0.511
I (%)	2 (6.1)	0 (0)	3 (7.9)	
II (%)	20 (60.6)	21 (60.0)	24 (63.2)	
III (%)	11 (33.3)	14 (40.0)	11 (2.9)	

*BMI* body mass index, *ASA* American Society of Anesthesiologists, *CEA* carcinoembryonic antigen

## Discussion

In this study, the learning curve of operation time can be divided into three phases. In phase 1 (cases 1 to 33), the surgeon began to become familiar with the manipulation of the robotic platform and started to establish the surgical procedures of right hemicolectomy. Thus, the operation time and intraoperative blood loss were higher than those in phases 2 and 3, and the slope of the learning curve was positive. In phase 2 (cases 34 to 68), because the surgical procedures were further optimized and cooperation with assistants was enhanced, the learning curve reached the plateau stage. In phase 3 (cases 69 to 106), along with the increased proficiency of robotic manipulation and cooperation with assistants, the surgical procedures reached the highest optimization. The surgeon mastered robotic right hemicolectomy, and the operation time and intraoperative blood loss during this phase were less than those of phases 1 and 2. Thus, the slope of the learning curve exhibited a declining trend.

However, a clear turning point was not seen on the RA-CUSUM curve in this study. Because surgical failure was rare in our cohort, the learning curve exhibited a continuous downward trend. The results indicated that robotic right hemicolectomy is relatively easy to master for surgeons with experience in laparoscopic surgery.

Because this study was based on retrospective data and only a single experienced laparoscopic surgeon, bias may exist, and the generalizability may be reduced. Future multicenter prospective studies are needed to demonstrate this hypothesis.
